# Light exposure before learning improves memory consolidation at night

**DOI:** 10.1038/srep15578

**Published:** 2015-10-23

**Authors:** Li-Li Shan, Hao Guo, Ning-Ning Song, Zheng-Ping Jia, Xin-Tian Hu, Jing-Fei Huang, Yu-Qiang Ding, Gal Richter-Levine, Qi-Xin Zhou, Lin Xu

**Affiliations:** 1 Key Laboratory of Animal Models and Human Disease Mechanisms, Kunming Institute of Zoology, the Chinese Academy of Science, Kunming 650223, China; 2 KIZ-SU Joint Laboratory of Animal Models and Drug Development, College of Pharmaceutical Sciences, Soochow University, Suzhou 215123, China; 3 Laboratory of Learning and Memory, Kunming Institute of Zoology, the Chinese Academy of Science, Kunming 650223, China; 4 University of the Chinese Academy of Sciences, Beijing 100049, China; 5 Collaborative Innovation Center for Brain Science, Department of Anatomy and Neurobiology, Tongji University School of Medicine, 1239 Siping Road, Shanghai 200092, China; 6 Neurosciences & Mental Health, The Hospital for Sick Children, Department of Physiology, Faculty of Medicine, University of Toronto, 555 University Ave., Toronto, Ontario 5MS 3H2, Canada; 7 CAS Center for Excellence in Brain Science, 320 Yue Yang Road, Shanghai, 200031, China; 8 State Key Laboratory of Genetic Resources and Evolution, Kunming Institute of Zoology, the Chinese Academy of Sciences, Kunming 650223, China; 9 The Institute for the Study of Affective Neuroscience, and Sagol Department of Neurobiology and Department of Psychology, University of Haifa, Haifa, Israel

## Abstract

Light is recently recognized as a modulator able to activate the hippocampus and modulate memory processing, but little is known about the molecular mechanisms. Here, we report that in mice, a short pulse of white light before learning dramatically improves consolidation of contextual fear memory during the night. The light exposure increases hippocampal active p21-activated kinase 1 (PAK1) and CA1 long-term potentiation (LTP). These light effects are abolished in PAK1 knockout and dominant-negative transgenic mice, but preserved by expression of constitutively active PAK1 in the hippocampus. Our results indicate that light can act as a switch of PAK1 activity that modulate CA1 LTP and thereby memory consolidation without affecting learning and short-term memory.

Light is essential for visual functions. Light also exerts non-imaging forming effects that can modulate many behaviors and physiological functions^1^,^2^,^3^. Several studies have demonstrated that short exposure to light significantly improves the brain responses to cognitive tasks^4^,^5^,^6^, such as declarative memory performance^7^, which is known to be closely associated with hippocampal function^8^, that triggers a greater activation of the hippocampus with the light exposure^2^,^6^,^9^,^10^. Nevertheless, little is known yet about the molecular and cellular mechanisms by which the hippocampus-dependent memory is regulated by light.

Newly formed memory can be stabilized by series of consolidation processing, in which the early phase is termed cellular consolidation or synaptic consolidation. Cellular consolidation is based on a pervasive concept that synaptic plasticity such as long-term potentiation (LTP) is critical for memory, and its maintenance enables the stabilization of newly formed memory into long-term memory (LTM)^11^,^12^,^13^. A number of studies reports that the local phosphorylation state of molecules in the synapses can act as a key switch to regulate LTP and LTM^14^,^15^,^16^,^17^,^18^,^19^. The p21-activated kinase 1 (PAK1) is a highly conserved serine-threonine kinase and plays critical role in synaptic plasticity and memory^20^,^21^,^22^,^23^. Transiently active PAK1 causes an enhanced and prolonged LTP in the hippocampus^20^,^21^. In mice, PAK1 knockout significantly impairs both hippocampal LTP and LTM^21^. Consistently, dominant-negative PAK1 transgenic mice express attenuated consolidation of the hippocampus-dependent spatial and fear memories although normal short-term memory (STM)^23^.

Here, we report for the first time that PAK1 activity can be modulated by light exposure, as a single pulse of light for 30 min significantly enhanced hippocampal PAK1 activity at night. In addition, using the hippocampus-dependent contextual fear conditioning (CFC) in mice, we found that the light treatment before but not after learning dramatically improved LTM at night, without any effect on learning and STM. Furthermore, the improved LTM was tightly associated with larger CA1 LTP, both of which were dependent on the enhanced hippocampal active PAK1 by the light exposure at night. Thus, our findings provide a new insight into the molecular and cellular basis by which the light exposure at night improves consolidation of memory specifically as there was no change in learning and STM.

## Results

### Light exposure enhances memory consolidation and PAK1 activity

**Figure 1:**
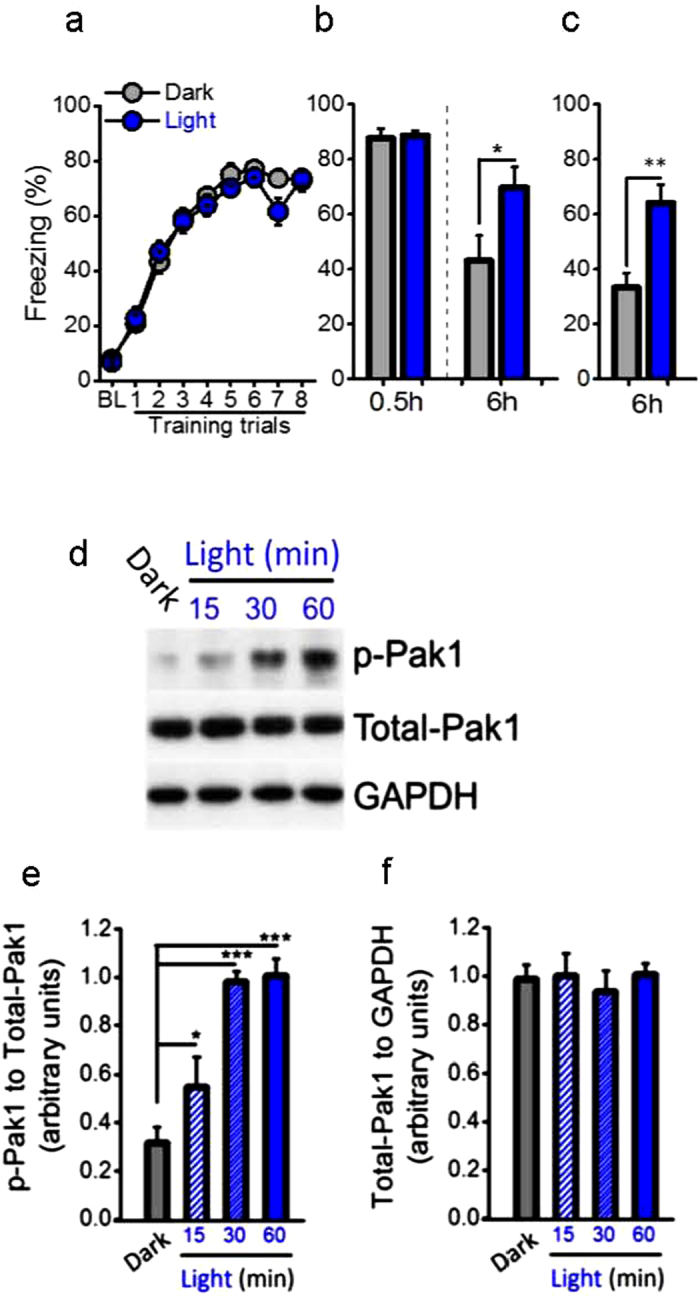
Light improves consolidation of memory and increases active PAK1 during the night. Contextual fear conditionings (CFC), 30 min short-term memory (STM) and 6 h long-term memory (LTM) were examined in C57BL/6 mice during the night. (**a**) The darkness and light treatment before CFC enabled similar learning (n = 27 per group; group, *F*_(1, 52)_ = 0.528, *P* = 0.471, repeated measured ANOVA) and (**b**) STM (0.5 h, n = 12 per group, *t*_*(22)*_ = 0.249, *P* = 0.805, *t* test). However, the light treatment improved significantly consolidation of memory at 6 h after CFC compared with control mice that were kept in darkness, in either with (6 h, n = 12 per group; *t*_*(22)*_ = 2.241, *P = 0.035, *t* test) (**b**) or (**c**) without STM test (6 h, n = 15 per group; *t*_*(22)*_ = 3.853, **P = 0.001, *t* test). (**d,e**) The down-regulated active PAK1 during the night was rapidly increased by the light treatment (n = 7 per group, light, *F*_*(1, 24)*_ = 32.379, ***P = 0.000, two-way ANOVA, the post-hoc test for Dark group compared to Light 15 min, 30 min and 60 min: *P* = 0.054, ***P = 0.000, ***P = 0.000) with a time-dependent effect (15–60 min) (time, *F*_*(2, 24)*_ = 10.291, **P = 0.001), but (**f**) no changes in total-PAK1 (n = 7 per group, interaction, *F*_*(3, 24)*_ = 0.192, *P* = 0.901, two-way ANOVA).

Given light acutely activating the hippocampus and modulating the hippocampus-dependent memories, we analyzed the roles of light on the hippocampus-dependent contextual fear conditioning. We found that mice expressed better memory during the day than during the night (Supplementary Fig. 1a–c), which is consistent with previous studies^24^,^25^. To exclude out circadian effects and investigate whether light exposure could influence the memory, the following experiments were strictly confined to the dark period, circadian time (CT) 19 to CT 7. Control mice were kept in darkness (<1 lux), while experimental mice received a 30 min light pulse starting at CT 21 by using the white light-emitting diodes (LEDs, ~300 lux inside cage) that were placed above homecage. After the light treatment, animals were subjected to CFC. STM at 30 min and subsequent LTM at 6 h after the CFC were measured. LTM was also measured in independent groups without STM test. Here we demonstrated that control mice displayed similar learning curve and STM (Fig. 1a,b) but showed a largely faded LTM, relative to the experimental group with the light treatment before the CFC (Fig. 1b,c; 6 h with STM test, *P* < 0.05; 6 h without STM test, *P* = 0.001). Thus, significantly better LTM is indeed reproduced by the light treatment at night. However, this was not observed when the light pulse was applied immediately after the CFC or before LTM test, as shown by similar freezing level compared to controls (Supplementary Fig. 2a–c, 6 h, *P* = 0.862). These results indicated that light before but not after learning dramatically improves memory consolidation.

To investigate the cellular mechanisms underlie better LTM by light exposure before learning and based on previous reports^26^,^27^,^28^, we speculated that the light treatment might selectively activate certain kinase that contributed to the conversion of the STM into the LTM. Because the evolutional conserved kinase PAK1 has a rich expression in the hippocampus and contributes to memory consolidation, we examined the catalytically active form of PAK1 (i.e. phosphorylated PAK1, p-PAK1). We found for the first time that the hippocampal PAK1 activity underwent light/dark shifting (Supplementary Fig. 1d–f), consistent with the behavioral result found during the day and night (Supplementary Fig. 1a–c). Next, to exclude out the possibility that this change of active PAK1 was due to the internal circadian clocks, which can drive circadian rhythms in the absence of light information, we examined the acute effect of the light exposure (a single pulse for 15, 30 or 60 min) on active PAK1 during the night (starting at CT 21). Notably, active PAK1 was rapidly up-regulated by a 15-min light pulse, and reached to the peak level with a 30-min light pulse (Fig. 1d,e), suggesting that external light rather internal clocks determine active PAK1 (Fig. 1d,f). In addition, this light-induced up-regulation of active PAK1 was no longer existed 30 min after turned off the light during the night (Supplementary Fig. 3a–d). Thus, hippocampal active PAK1 is exquisitely sensitive to the light and dark condition at night.

### Inhibition of hippocampal PAK1 activity blocks light-enhanced LTM

**Figure 2:**
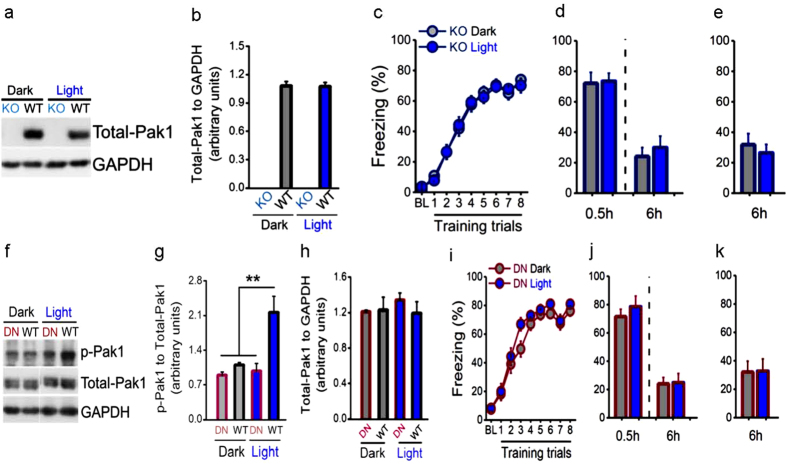
Genetically inhibiting PAK1 activity block light effect on memory. (**a,b**) Total-PAK1 was undetectable in KO mice in contrast to that in WT mice (n = 4 per group). (**c,d**) The light treatment before learning had no effects on learning (Light, n = 27, Dark, n = 24; group, *F*_(1, 49)_ = 0.005, *P* = 0.946, repeated measured ANOVA), STM (0.5 h, Light, n = 12, Dark, n = 15; *t*_*(25)*_ = 0.143, *P* = 0.888, *t* test) and LTM (6 h, Light, n = 12, Dark, n = 15; *t*_*(25)*_ = 0.558, *P* = 0.582, *t* test), and (**e**) LTM without STM test (6 h, n = 12 per group; *t*_*(22)*_ = 0.608, *P* = 0.550, *t* test), in relative to those of Dark group in KO mice. (**f,g**) Active PAK1 was at a very low level and not responsive to the light treatment in DN mice, but it was significantly increased by the light treatment in WT mice (n = 4 per group; *F*_(3, 15)_ = 10.331, **P = 0.001, one-way ANOVA, the post-hoc test for WT Light group compared to DN Dark, WT Dark and DN Light: ***P = 0.000, **P = 0.002, **P = 0.001), and (**h**) total-PAK1 was unaffected (*F*_(3, 15)_ = 0.400, *P* = 0.756, one-way ANOVA). (**i,j**) Similar to KO mice, the light treatment produced no effects on learning (Light, n = 24, Dark, n = 26; group, *F*_(1, 48)_ = 1.664, *P* = 0.203, repeated measured ANOVA), STM (0.5 h, Light, n = 12, Dark, n = 14; *t*_*(24)*_ = 0.783, *P* = 0.441, *t* test) and LTM (6 h, Light, n = 12, Dark, n = 14; *t*_*(24)*_ = 0.102, *P* = 0.920, *t* test), and (**k**) LTM without STM test (6 h, n = 12 per group; *t*_*(22)*_ = 0.053, *P* = 0.959, *t* test) compared to those of Dark group in DN mice.

Based on the above findings, we next examined the necessity of PAK1 activity in improving memory consolidation by the light exposure, by using PAK1 knockout (KO) mice. As expected, PAK1 was undetectable (Fig. 2a,b) and the light treatment failed to improve LTM in the KO mice (6 h, Fig. 2d,e), while no changes in learning curve (Fig. 2c) and STM (0.5 h, Fig. 2d). To further confirm our findings, we used the postnatal forebrain-specific PAK dominant negative (DN) transgenic mice, in which the endogenous PAK activity is largely suppressed^29^. We found that hippocampal active PAK1 in the DN mice was very low and not responsive to the light treatment in relative to WT mice, and total-PAK1 was not different (Fig. 2f–h). Importantly, the enhancement of LTM by the light treatment were absent in the DN mice (6 h, Fig. 2j,k), although the CFC induced similar learning curve (Fig. 2i) and STM (0.5 h, Fig. 2j). Thus, PAK1 activity in the hippocampus was necessary for improvement of memory consolidation by the light exposure at night.

### Increased hippocampal PAK1 activity mimics light effect on LTM

**Figure 3:**
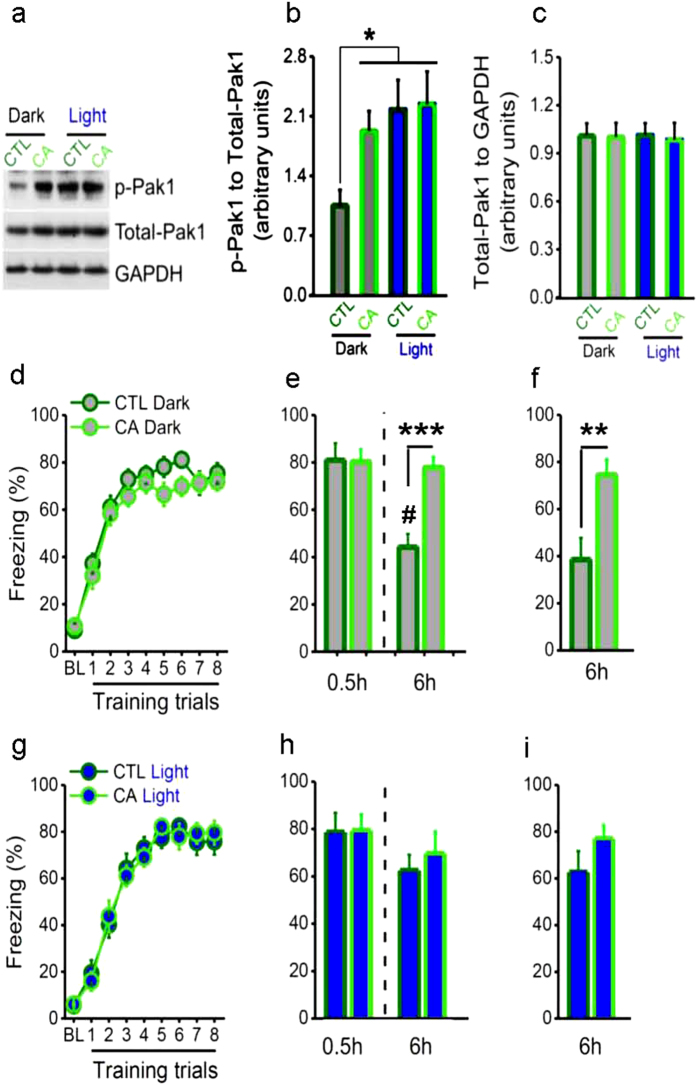
Constitutively increasing PAK1 activity enhances memory without light. The constitutively active PAK1 (CA) or eGFP virus (CTL) was expressed into bilateral CA1 regions of C57BL/6 mice. (**a,b**) Active PAK1 was significantly higher in CA than in CTL mice under dark condition, while active PAK1 was significantly increased in CTL mice but not in CA mice with the light treatment (n = 6 per group; *F*_(3, 20)_ = 3.385, *P = 0.038, one-way ANOVA, the post-hoc test for WT Dark compared to CA Dark, WT Light and CA Light: *P = 0.048, *P = 0.015 and *P = 0.011), and (**c**) no changes in total-PAK1. (**d,e**) Without the light treatment, learning curve (CA, n = 24, CTL, n = 23; virus, *F*_(1, 45)_ = 1.516, *P* = 0.225, repeated measured ANOVA) and STM (0.5 h, CA, n = 12, CTL, n = 11) were similar in CA and CTL mice, but LTM was significantly better in CA than in CTL mice, with (6 h, *t*_(21)_ = 4.761, ***P = 0.000, *t* test) or (**f**) without STM test (6 h, n = 12 per group; *t*_(22)_ = 3.204, **P = 0.004, *t* test). (**e**) A significant decrement of LTM compared with STM in CTL mice but not in CA mice (CTL, *t*_(10)_ = 5.844, ^#^*P* < 0.001; CA, *t*_(11)_ = 0.415, *P* = 0.686, *t* test). (**g,h**) The light treatment enabled similar learning (CA, n = 18, CTL, n = 17; virus, *F*_(1, 33)_ = 0.003, *P* = 0.956) and STM (0.5 h, n = 8 per group), while it enhanced LTM in CTL mice but failed to do so in CA mice, with (6 h, *t*_(14)_ = 0.501, *P* = 0.624, *t* test) or (**i**) without STM test, and resulted in no significant difference between the groups (CA, n = 10, CTL, n = 9; *t*_(17)_ = 1.250, *P* = 0.228, *t* test ). (h) No significant difference between LTM and STM in both CTL mice and CA mice (CTL, *t*_(7)_ = 1.107, *P* = 0.305; CA, *t*_(7)_ = 0.608, *P* = 0.562, *t* test).

To further confirm active PAK1’s role in light-enhanced memory consolidation, we used adeno-associated virus (AAV) to express the constitutively active (CA, T423E) PAK1 (AAV2-CMV-CA-PAK1-eGFP, CA group) or eGFP (AAV2-CMV-eGFP, CTL group) into the bilateral hippocampal CA1 regions of adult mice (Supplementary Fig. 4). In the CA mice, hippocampal active PAK1 displayed a higher level relative to the CTL group, and unlike the CTL mice active PAK1 could not be up-regulated by the light treatment, probably due to the ceiling effect that may occlude the addition of the light-increased active PAK1 (Fig. 3a,b). Total-PAK1 was not obviously changed in the CA mice relative to the CTL mice (Fig. 3c). Interestingly, without the light treatment these two groups of mice showed similar learning curve and STM (Fig. 3d,e), but LTM was significantly enhanced in the CA mice (Fig. 3e,f). On the other hand, the light treatment was able to improve LTM in the CTL mice, but failed to do so in the CA mice as revealed by unchanged freezing level relative to those without the light treatment (Fig. 3g–i, Dark vs. Light, 6 h with STM test, *P* = 0.425; 6 h without STM test, *P* = 0.832). This is probably due to the ceiling effect of active PAK1 on memory consolidation in the CA mice. In addition, there was a significant decrease of LTM compared to STM in the CTL group but not in the CA group without light treatment (Fig. 3e). This decrement also did not exist in the CTL group with the light exposure (Fig. 3h), suggesting that enhancement of hippocampal PAK1 activity in the CA group even under dark condition can mimic the improvement of LTM by the light treatment, strongly suggesting the sufficiency of active PAK1 in memory consolidation. Thus, we demonstrate for the first time that the light treatment before learning increases hippocampal PAK1 activity that is essential for better memory at night.

### PAK1 activity is essential for light-enhanced LTP

**Figure 4:**
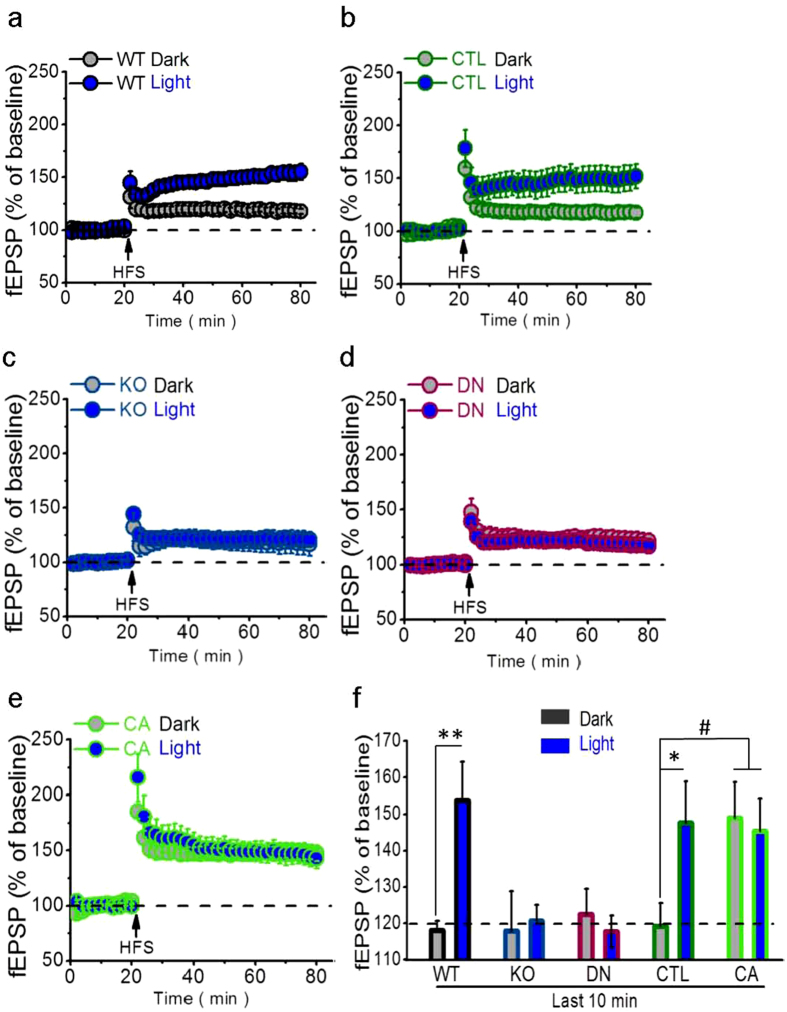
Light-enhanced LTP requiring hippocampal PAK1 activity. (**a,b**) In C57BL/6 mice, high-frequency stimulation (HFS) induced significantly larger CA1 LTP with the light treatment (WT, n = 13 slices; CTL, n = 7 slices) than under darkness (WT, n = 12 slices; CTL, n = 8) before slice preparation in both WT (*F*_(1, 23)_ = 10.563, **P = 0.004, repeated measured ANOVA) and CTL mice (*F*_(1, 13)_ = 6.169, *P = 0.027, repeated measured ANOVA) (WT, n = 6 mice per group; CTL, n = 3 mice per group). However, (**c,d**) regardless of darkness (KO, n = 13 slices; DN, n = 12 slices) or light treatment (KO, n = 10 slices; DN, n = 12 slices) before slice preparation, HFS similarly induced smaller CA1 LTP in both KO (Dark, n = 6 mice; Light, n = 5 mice; *F*_(1, 21)_ = 0.052, *P* = 0.822, repeated measured ANOVA) and DN mice (Dark and Light, n = 6 mice per group; *F*_(1, 22)_ = 0.316, *P* = 0.580, repeated measured ANOVA). In marked contrast, (**e**) No matter darkness (n = 7 slices) or light treatment (n = 9 slices) before slice preparation, HFS similarly induced larger CA1 LTP in CA mice (Dark, n = 4 mice; Light, n = 5 mice; *F*_(1, 14)_ = 0.065, *P* = 0.803, repeated measured ANOVA). (**f**) The LTPs were significantly larger with the light treatment in WT/CTL or CA-PAK1 groups than in darkness or PAK1-KO/DN groups (*F*_(1, 102)_ = 3.661, **P = 0.001, ANOVA, the post-hoc test for WT Dark compared to WT Light, **P = 0.001 and CTL Dark compared to CTL Light, CA Dark and CA Light, *P = 0.019 , ^*#*^*P* = 0.023 and ^*#*^*P* = 0.031).

Because the increased active PAK1 by the light pulse has a short-lifetime as it was rapidly down-regulated 30 min after turned off the light (Supplementary Fig. 3b,c), we inferred that the light treatment through up-regulation of active PAK1 might had acted as a molecular switch to influence hippocampal LTP induction and thus contributed to memory consolidation. Thus, we next examined LTP induction in the Schaffer-CA1 synapses *in vitro* using high-frequency stimulation (HFS). Slices were prepared from experimental mice immediately after the light treatment (CT 21) or from control mice kept in darkness. In both WT and CTL mice, relative to darkness the light treatment enabled a larger LTP induction (Fig. 4a,b). In marked contrast, the light treatment did not endow a large LTP in KO or DN mice as shown by no significant difference compared to that of dark group (Fig. 4c,d). This finding is consistent with previous reports in KO mice^20^,^21^,^30^, but not in DN mice in which LTP is enhanced by using theta burst stimulation^29^ probably due to a notion that different LTP induction protocol and brain regions might be attributable^31^. Conversely, a larger LTP was observed in CA mice regardless of the light treatment or not (Fig. 4e). These data support the idea that the light treatment-increased hippocampal active PAK1 engaged in larger LTP, and thereby better memory (Fig. 4f, small *vs*. large LTP, *P* < 0.001).

## Discussion

Previous studies have focused on whether light exposure can increase activation of certain brain regions during cognitive tasks^4^,^5^,^6^,^7^,^32^. Also, ample study examined the neural network through which light information is conveyed to subcortical structures and limbic areas^2^,^6^,^9^,^10^. These non-imaging forming effects of light may have profound influences on cognitions, but little is known yet about the molecular and cellular mechanisms that underlie modulation of memory processing by light exposure. Here, our study provided a new insight into the mechanisms by which the hippocampus-dependent memory was improved by light exposure at night. The mechanisms could be very complex, but we provided evidence here that hippocampal active PAK1 was critical, as a key modulator responsive to light exposure at night that led to larger CA1 LTP and better consolidation of memory.

It was unclear why the light exposure before but not after learning had a profound impact on memory consolidation. First, we believe that the timing between active PAK1 and CFC is critical because active PAK1 can regulate the induction of CA1 LTP, which is widely believed to be the underlying mechanism for memory. Second, active PAK1 in the synapses that occurred LTP may enable a better stabilization of the memory trace so that improved memory consolidation. Without the active PAK1 in the selected synapses during learning or the induction of CA1 LTP, the memory trace could be rapidly decayed. This could be a reasonable explanation for why the light exposure only before learning can improve consolidation of memory without affecting learning and STM. Therefore, PAK1 activity plays a central role in effects of the light exposure at night. In agreement with our opinion, using PAK1 KO and DN mice or constitutively active PAK1 expression in the hippocampus, we demonstrated that PAK1 activity was indeed both necessary and sufficient for improvement of memory consolidation by the light exposure at night. Taken together, we suggest that PAK1 activity, the short-lifetime state of phosphorylation, plays as a key role in memory consolidation particularly important at night when PAK1 activity is down-regulated but can be up-regulated by a short period of light exposure.

Our novel finding could be also important to understand why some STMs are converted to LTMs but others do not. It is possible that active PAK1 could be rapidly increased in the selected synapses where the memory trace is able to be placed by strengthening of the synaptic efficacy. Consistent with our LTP study in the hippocampus, we found that active PAK1 was also necessary and sufficient for a larger CA1 LTP. Therefore, our findings are in accordance with the pervasive notion that LTP is the underlying mechanism for memory^11^,^12^,^13^, and this mechanism is exquisitely sensitive to active PAK1 that is entrainable to light exposure.

Furthermore, the fact that a short pulse of light exposure before but not after learning can improve memory consolidation could strongly support a cellular consolidation mechanism proposed by synaptic tagging and capture hypothesis^26^, consistent to the finding that novelty exposure before learning leads to better LTM^33^. Recently, several studies report that certain kinases can enable the synapses being a tagged state that benefits LTM without influence STM^27^,^28^,^33^,^34^. Therefore, our finding supports the notion that memory consolidation can be predetermined by a tagged state of the synapses that is sensitive to environmental stimuli such as novelty and light exposure etc. Here, we demonstrate for the first time that PAK1 activity could be one of the labels for synaptic tagging, which can be marked by a short pulse of light at night.

Our findings also support a mechanism for understanding the cycling of cognition during the day and night^25^, as PAK1 activity and memory consolidation follow a day/night pattern (Supplementary Fig. 1d–f). Notably, our finding highlights a possible basis for the clinically used light therapy that is applied at night to alleviate memory loss in Alzheimer’s disease^35^,^36^.

## Methods

### Animals

The Swiss (or Kunming) mice were purchased from the Animal House Center, Kunming Medical University, Kunming, China. The C57BL/6 mice were purchased from the Vital River Laboratory Animal Technology Co. Ltd., Beijing, China. The *PAK1* knockout (KO) mice^21^ with the mixed C57BL/6 and strain 129 genic background were donated by Prof. Zhengping Jia from University of Toronto, Canada. The *dnPAK* transgenic mice (C57BL/6-Tg (Camk2a-AIDPAK) 21 Stl/J)^37^ were purchased from the Jackson Laboratory through Prof. Yuqiang Ding’s Laboratory from Tongji University, China. Male mice aged 2–4 months were used. Mice were housed 2–4 per cage, free access to food and water, under regulated room temperature, and with a 12 h light and 12 h dark cycle (light on at CT 7) for at least 7 d^24^ in an Animal Facilitate of Kunming Institute of Zoology, the Chinese Academy of Sciences. All experiments were performed at night under dark condition (<1 lux) except the time period when the light treatment (300 lux) for 30 min or 3 h was applied. Animal care and experimental procedures were, in accordance with the Institutional Guidelines, reviewed and approved by the Animal Ethics Committee of Kunming Institute of Zoology, the Chinese Academy of Sciences.

The Swiss mice, because of locally available (Kunming), were used in Supplementary Fig. 1 as a preliminary study. The C57BL/6 mice (air transportation from Beijing) were then used in all of the other studies. LTM examined 6 h after CFC (in the same dark cycle) is to avoid any influences possibly induced by switching of the light/dark environment.

### Light treatment or dark condition during the night

The white light-emitting diodes (LEDs) were applied directly above homecage. The light treatment led to an illumination more than 300 lux inside the homecage^38^. In contrast, without the light treatment, animals were kept in a dark condition with an illumination less than 1 lux^24^.

### Contextual fear conditioning

The procedure for contextual fear conditioning (CFC) was modified from those described previously^39^,^40^,^41^,^42^,^43^. The training chambers of CFC were purchased from the Med Associates with a Near Infrared Video System, which is supported by a near infrared camera (Med Associates Inc., Vermont, USA). Before CFC, mice were allowed to acclimate to the chambers without footshock for continuous 3 d. The CFC paradigm was consisted of a 5 min measurement for baseline freezing level (BL) and 8 training trials with electrical footshock (3 s, 1.2 mA) and the average of intertrial intervals was about 2 min. The mice were placed back to homecage 2 min after the final trial. The chambers were cleaned by using 25% ethanol before training. Then, short-term memory (STM) 30 min and/or long-term memory (LTM) 6 h after the CFC were measured by placing animals back to the conditioned chambers for 3 min. All experiments were performed under the dark condition (<1 lux) during the night except the light treatment mentioned elsewhere. The freezing behavior was monitored by the Video System and stored in a computer. The freezing level that indicates the performance of learning and memory was automatically measured by the software from Med Associates Inc.

### Western blot analyses

The paradigm used was similar to those described previously. Mouse hippocampus was frozen in liquid nitrogen and homogenized in RIPA buffer (Beyotime Biotech.) added with 1mmol PMSF. Samples were mixed (3:1) with the 4 × SDS loading buffer (250 mmol Tris-Hcl, pH 6.8, 20% ß-mercaptoethanol, 4% SDS, 0.004% bromophenol blue (wt/vol), 40% (vol/vol) glycerol), and denatured by boiling for 10 min at 100 °C. Each sample was run on a SDS-PAGE (Bio-Rad) and transferred to a PVDF membrane (Millipore). Blots were blocked at room temperature with block buffer (Millipore). For western analysis, we used rabbit polyclonal antibody was to p-PAK1 Thr 423 antibody (1:1000, Cell Signaling) and PAK1 antibody (1:1000, Cell Signaling), and mouse monoclonal antibody to GAPDH (1:20000). When necessary, blots were stripped with stripping buffer (0.1% SDS (wt/vol), 1% Tween−20 (vol/vol), 1.5% Glycine (wt/vol)). Immunoreactivity was detected using luminata crescendo western HRP substrate (Millipore), and net intensity values were determined using the Image J software and were normalized to total GAPDH.

### Electrophysiology

Brain coronal slices were prepared from these types of mice with the dark or light treatment similar to behavioral protocols during the night by using protocols describe previously^39^. Brains were dissected in ice-cold artificial cerebrospinal fluid (ACSF) and cut (400-μm-thick) with a Leica VT 1000 vibratome (Leica Biosystems) at 0 °C. Before being transferred to a submerged recording chamber, slices were recovered for at least 30 min in oxygenated (95% O_2_ and 5% CO_2_) warm (37 °C) ACSF containing (in mmol) 120 NaCl, 2.5 KCl, 2 CaCl_2_, 2 MgSO_4_, 26 NaHCO_3_, 1.25 NaH_2_PO_4_, 10 glucose. Hippocampal CA1 LTP experiment were recording at room temperature, and was induced by three trains of HFS (high frequency stimulation, each with 1 pulse at 100 hertz for 1 s) delivered at 30 s intervals. Recording electrodes (4–6 MΩ) were pulled on a Micropipette Puller (Sutter Instruments, California, USA).

LTP was measured by using the averaged amplitude of the fEPSP during the last 10 min recordings^39^. Data were acquired by using a Multiclamp 700 B amplifier connected to a Digidata 1440 analog of the digital converter. All recordings were digitized and analyzed using pClamp 10.0 software (Axon Instruments Inc., California, USA).

### Viral Administration

Mice were anesthetized with pentobarbital sodium (8 mg per kg; i.p.) and the scalp was shaved. After being mounted on a stereotaxic apparatus (RWD Life Science Co., Shenzhen, China), four small holes were drilled to allow for viral injections into both the hemispheres of the hippocampal CA1 areas.

The pAOV.eGFP.PAK1 or pAOV.eGFP cDNAs were subcloned under the AAV 2/8 coexpresses eGFP driven by a CMV promoter (Neuron Biotech Co., Ltd, Shanghai, China) as those described^29^. A 0.5 μl of high-titer (>5.0 × 10^12^ v.g./ml) AAVs was administrated per site. The coordinates for stereotaxic injections were −2.5 mm anterior-posterior, ±2 mm medial-lateral, −1.2 mm dorso-ventral from bregma for one site and −1.5 mm, ±1 mm, −1.2 mm for another at a rate of 0.1 μ/min. Injection needles were left in the holes for 5 min post administration to make sure distribution of the viruses.

### Tissue Preparation

After AAV viral injection for 4 weeks, mice were anesthetized with pelltobarbitalum natricum and transcardially perfused with 0.9% NaCl followed by 4% paraformaldehyde (PFA 4%) dissolved in 0.1 mol phosphate buffered saline (PBS 0.1 mol). Brains were extracted and post fixed in PFA 4% for 24 h. Brains were transferred to 15% sucrose for 48–72 h and then to 30% sucrose for 48–72 h before slicing 20 μm coronal frozen sections of the entire hippocampus. Sections were mounted on slides and cover slipped using mounting media with DAPI (H-1200, Vector) to label cell nuclei and stored at 4 °C.

### Confocal Microscopy

A confocal laser scanning microscope was used for all image acquisition (FV 1000, Olympus). Images of cells expressing eGFP and DAPI were collected for the hippocampal CA1 by using a 10 × objective and a 20 × objective.

### Statistical Analysis

The data were analyzed by one- or two-way ANOVA or *t* test, including repeated measures ANOVA whenever appropriate. Using Kolmogorov-Smirnov tested normal distribution. Error bars were displayed as mean ± S.E.M., the criterion for statistical significance was *P < 0.5, **P < 0.01, ***P < 0.001.

## Additional Information

**How to cite this article**: Shan, L.-L. *et al*. Light exposure before learning improves memory consolidation at night. *Sci. Rep*. **5**, 15578; doi: 10.1038/srep15578 (2015).

## Supplementary Material

Supplementary Information
