# Corrigendum: Light exposure before learning improves memory consolidation at night

**DOI:** 10.1038/srep19172

**Published:** 2016-01-20

**Authors:** Li-Li Shan, Hao Guo, Ning-Ning Song, Zheng-Ping Jia, Xin-Tian Hu, Jing-Fei Huang, Yu-Qiang Ding, Gal Richter-Levin, Qi-Xin Zhou, Lin Xu

Scientific Reports 5: Article number: 15578; 10.1038/srep15578 published online: 10232015; updated: 01202016.

The original version of this Article contained a typographical error in the spelling of the author Gal Richter-Levin, which was incorrectly given as Gal Richter-Levine. This has now been corrected in both the PDF and HTML versions of the Article.

