# Supplementary material for: Light exposure before learning improves memory consolidation at night
Source: Sci Rep. 2015 Oct 23;5:15578. doi: 10.1038/srep15578 (PMC4616152; doi:10.1038/srep15578)
Supplement: Supplementary Information [file srep15578-s1.doc]

**Supplementary Materials for**

**Light exposure before learning improves memory consolidation at night**

Li-Li Shan^a-d^, Hao Guo^a,c,d^, Ning-Ning Song^e^, Zheng-Ping Jia^f^, Xin-Tian Hu^a,g^, Jing-Fei Huang^b,h^, Yu-Qiang Ding^e^, Gal Richter-Levine^i^, Qi-Xin Zhou^a-c,1^, and Lin Xu^a-d,g,1^

^1^To whom correspondence should be addressed. E-mail: [qixin_zhou@126.com](mailto:qixin_zhou@126.com) or [lxu@vip.163.com](mailto:lxu@vip.163.com)

**Supplementary Figures 1 to 4**

**
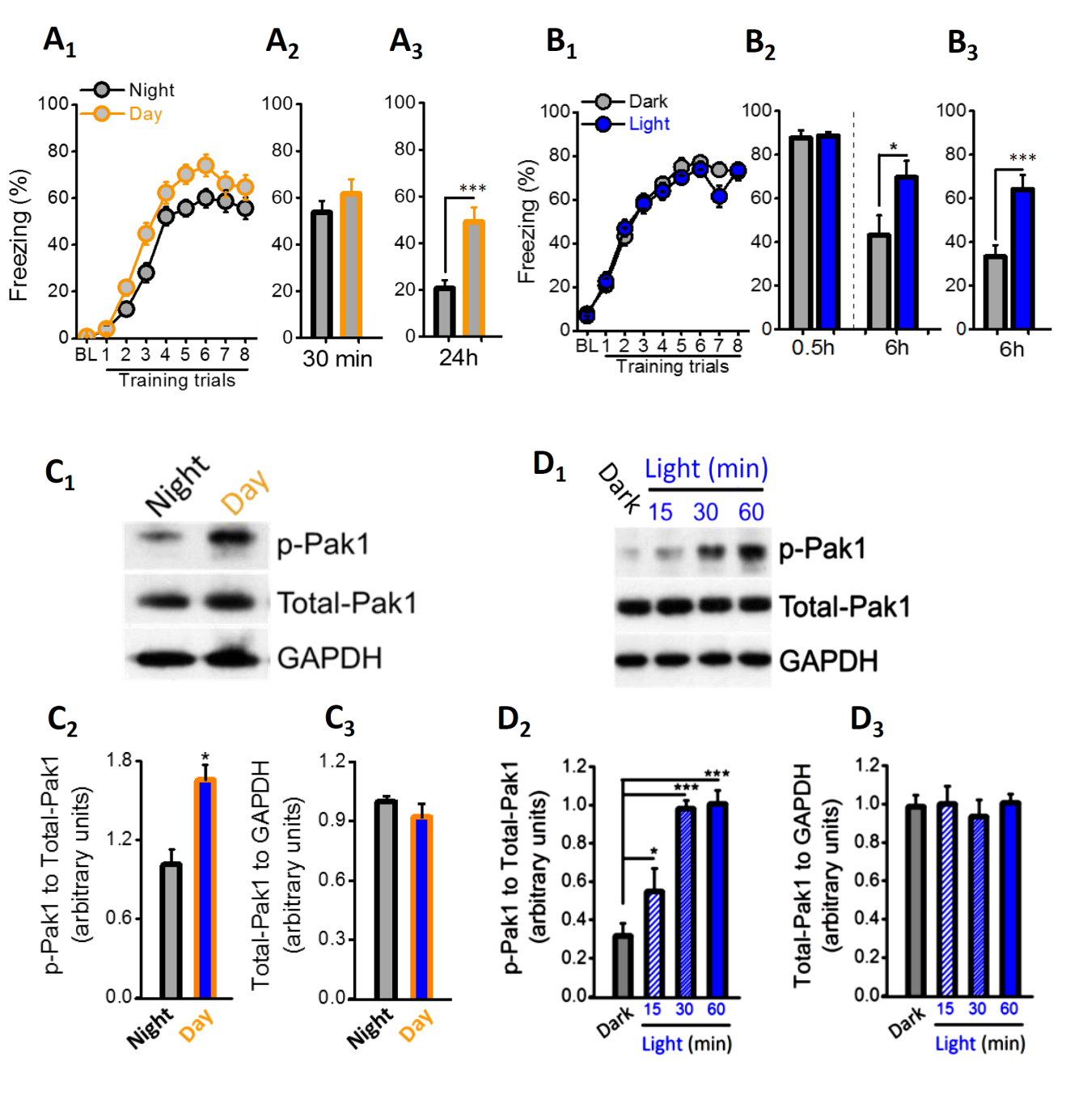
**

a b c

d

e f

**
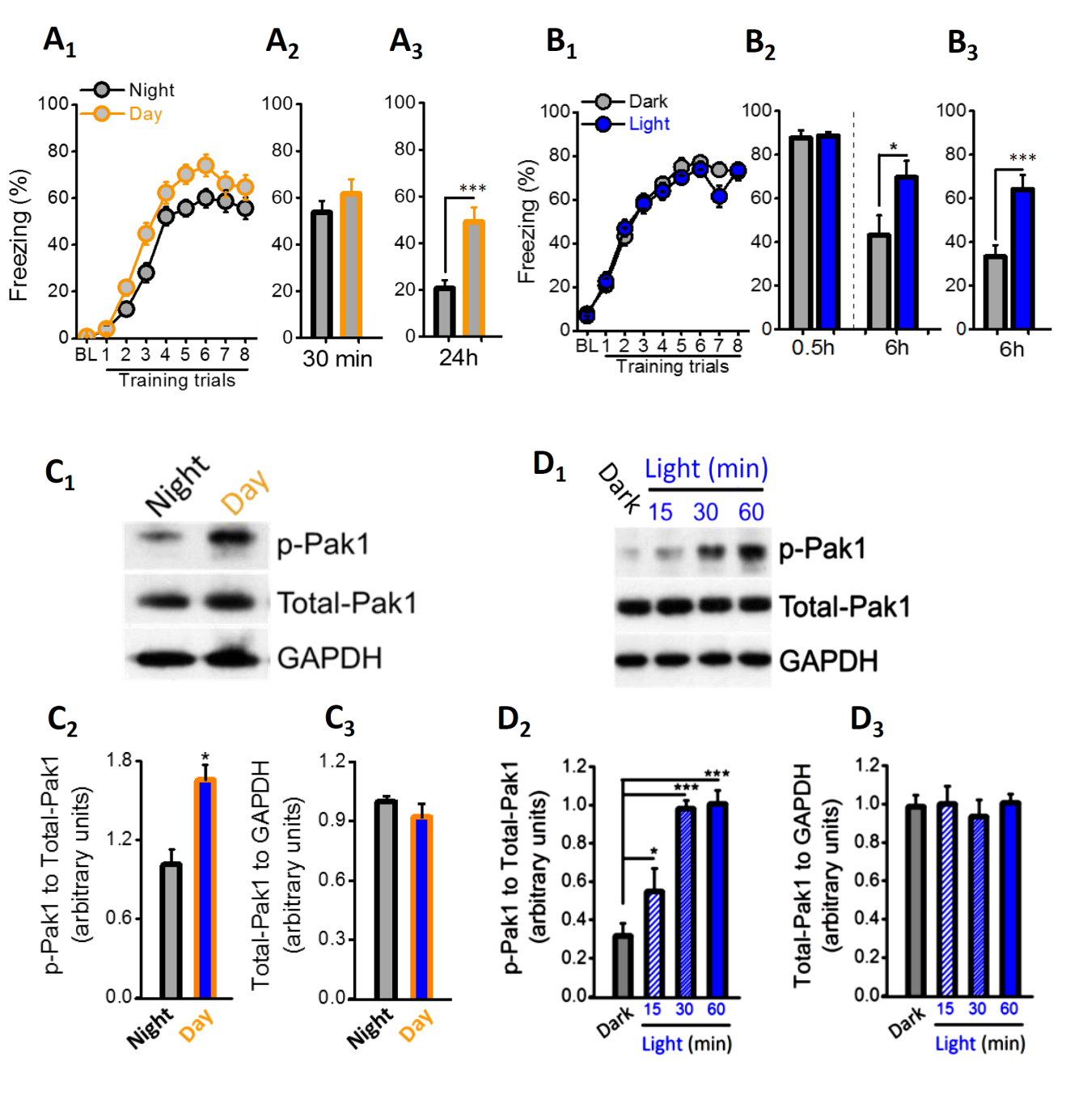
**

**Supplementary Figure 1 | LTM and PAK1 activity was greater during the day than during the night.** Contextual fear conditioning (CFC), 30 min short-term memory (STM) and 24 h long-term memory (LTM) were examined during the day at CT 12 (Day, n = 34) or during the night at CT 24 (Night, n = 45) in Swiss mice. (a) Although the both groups showed similar learning curve (trial, *F*_(6, 77)_ = 136.523, *P* = 0.000; interaction, *F*_(6, 77)_ = 1.586, *P* = 0.149, repeated measured ANOVA) and (b) STM (Night, n = 18; Day, n = 15; 0.5 h, *t*_(31)_ =1.018, *P* =0.316, *t* test), (**c**) the Day group exhibited significantly better 24 h LTM than the Night group (Night, n = 27; Day, n = 19; 24 h, *t*_(44)_ =4.325, ****P* = 0.000). (d-e) Active PAK1 (or phosphorylation of PAK1, p-PAK1) was significantly higher at CT 12 than at CT 24 (n = 3 per group, *t*_(4)_ = 3.892, **P* = 0.018, *t* test), (d, f) while the total fraction of PAK1 (total-PAK1) was unchanged (*t*_(4)_ = 1.096, *P* = 0.335).

a

c

b

**Supplementary Figure 2 | Light exposure after learning had no effects on memory consolidation.** A pulse of the blue-enriched white light for 3 h was applied above homecages after contextual fear conditioning (CFC) and long-term memory (LTM) at 6 h after learning (6 h RT) was tested during the night by using C57BL/6 mice. (a-c) Animals were divided into three groups after CFC randomly. Control mice (Dark/Dark, n = 17) were kept in darkness for 6 h, while the other two groups were exposed to the light pulse either immediately after CFC (Light/Dark, n = 10) or before LTM test (Dark/Light, n = 10). These groups showed no significant differences in LTM (*F* _(2, 34)_ = 0.149, *P* = 0.862, one-way ANOVA).


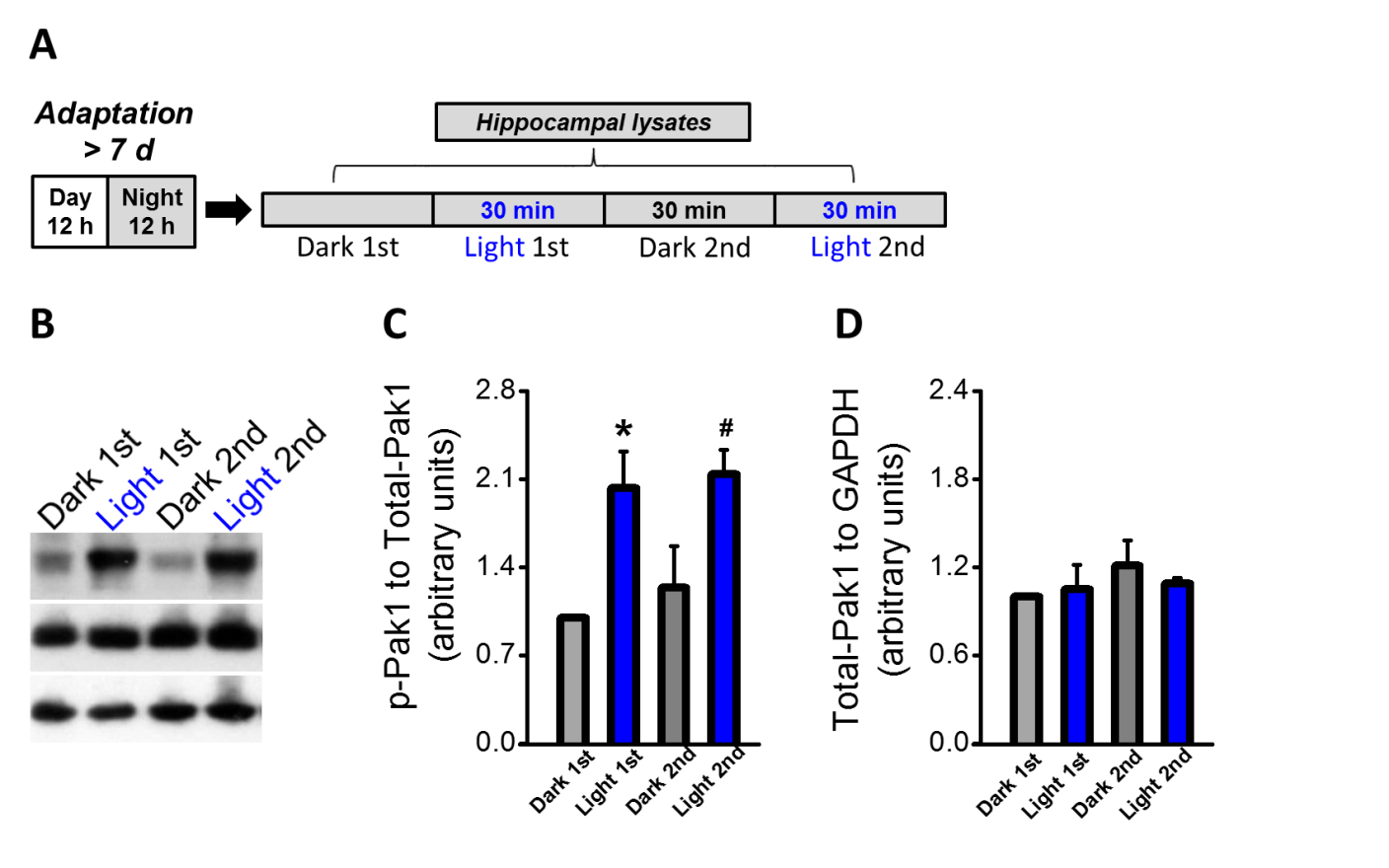


**Supplementary Figure 3 | Hippocampal PAK1 activity was regulated by light on/off during the night.** (a) Schematic procedure. Hippocampal lysates were prepared from the animals at the end of each treatment (n = 3 per group) by using C57BL/6 mice. (b-d) Active PAK1 was significantly up-regulated by the light treatment (*F* _(3, 11)_ = 5.446, *P* = 0.025, ANOVA). The post-hoc test showed that active PAK1 was up-regulated by the first light treatment compared with the first darkness (**P* = 0.017, Dark 1st vs. Light 1st), but the up-regulated active PAK1 was rapidly down-regulated by turned off the light for 30 min (*P* = 0.051, Light 1st vs. Dark 2nd), to a level without difference from the first darkness (*P* = 0.505, Dark 1st vs. Dark 2nd). The second light treatment was still able to up-regulate active PAK1 (^#^*P* = 0.031, Dark 2nd vs. Light 2nd). Total-PAK1 was unaffected by the light treatment (*F* _(3, 11)_ = 0.549, *P* = 0.663).

a

b

c

d

a b

***
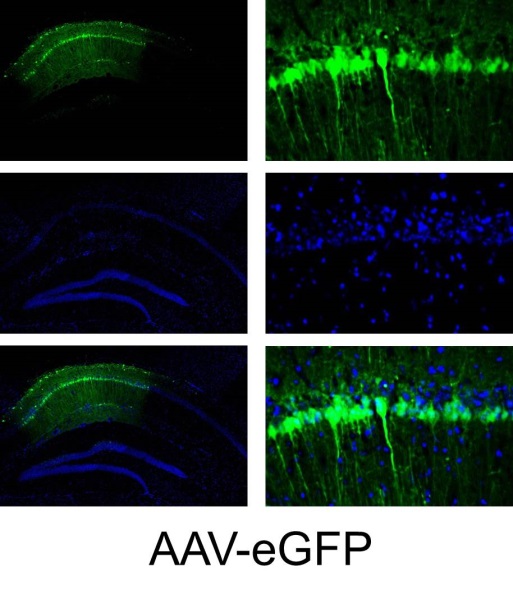

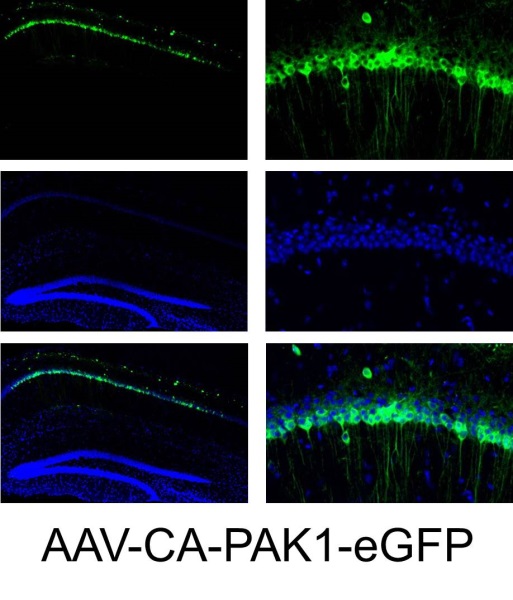
***

**Supplementary Figure 4 | Viral expression in hippocampal CA1 regions.** The constitutively active (CA) PAK1 was expressed into bilateral CA1 regions of adult C57BL/6 mice by using AAV-CMV-CA-PAK1-eGFP (CA group) and its control AAV-CMV-eGFP (CTL group). After behavioral experiments, hippocampal slices were prepared, and images were taken from CA1 regions using a confocal microscope. (a) A representative image from CA1 region of the mice injected with AAV-CMV-CA-PAK1-eGFP. (b) A representative image from CA1 region of the mice injected with AAV-CMV-eGFP**.**
